# Editorial: Fabrication of in-vitro 3D human tissue models—From cell processing to advanced manufacturing

**DOI:** 10.3389/fbioe.2022.1035601

**Published:** 2022-09-26

**Authors:** Wei Long Ng, May Win Naing, Ratima Suntornnond, Sanjairaj Vijayavenkataraman

**Affiliations:** ^1^ HP-NTU Digital Manufacturing Corporate Lab, Nanyang Technological University (NTU), Singapore, Singapore; ^2^ Singapore Institute of Manufacturing Technology (SIMTech), Agency for Science, Technology and Research (A*STAR), Singapore, Singapore; ^3^ Biomanufacturing Technology, Bioprocessing Technology Institute (BTI), Agency for Science, Technology, and Research (A*STAR), Singapore, Singapore; ^4^ The Vijay Lab, Division of Engineering, New York University Abu Dhabi, Abu Dhabi, United Arab Emirates; ^5^ Department of Mechanical and Aerospace Engineering, Tandon School of Engineering, New York University, Brooklyn, NY, United States

**Keywords:** 3D bioprinting, biofabrication, bio-inks, in-vitro 3D tissue models, cell expansion, microcarriers

Over the years, the field of toxicology testing has pivoted from the use of animal models to 2D human cell cultures and finally the adaptation of *in-vitro* 3D human testing models. It is important that these *in-vitro* 3D human testing models predicts the human responses in an accurate and reliable manner (Nam et al., 2015; Ng and Yeong, 2019). This is largely driven by the significant discrepancies between adverse effects of chemicals in humans and animals (Lilienblum et al., 2008). The use of animal models has several caveats which include the differences in the absorption or distribution of the chemicals/substances; the way the substances are metabolized and the short duration of animal lifespan to accurately monitor disease development. Similarly, conventional 2D cell culture is unable to adequately recapitulate the *in vivo* cell-cell and cell-matrix interactions found in native three-dimensional (3D) tissues and it has been reported that numerous types of cells have expressed different phenotypes and genomic profiles in 2D versus 3D cell culture (Duval et al., 2017; Jensen and Teng, 2020).

Hence, *in-vitro* 3D human tissue models would bring about the necessary complexity that may improve the reliability and accuracy of test outcomes. Some of the fabricated *in-vitro* 3D human tissue models for various testing applications include skin tissue models (Ng et al., 2018; Liu et al., 2020; Zhang et al., 2021), alveolar lung tissue models (Klein et al., 2013; Costa et al., 2019; Ng et al., 2021) and liver tissue models (Lee et al., 2015; Skardal et al., 2015; Hiller et al., 2018). However, there are some challenges faced in translating this to widespread use. Successful production of *in-vitro* tissue models is dependent on two critical aspects–ability to carry out large-scale manufacturing of cells (growing cells in vast quantities within a homogeneous physical and chemical environment) (Jordan et al., 2018; He et al., 2019; Chen et al., 2022) and advanced manufacturing platforms (highly-automated fabrication of *in-vitro* tissue models with high throughput rates and repeatability) (Ozbolat and Hospodiuk, 2016; Ng et al., 2019; Zhuang et al., 2019; Ng et al., 2020a; Ng et al., 2020b; Li et al., 2020; Ng et al., 2022; Suntornnond et al., 2022).

The goal of this Research Topic is to focus on the recent developments in cell processing techniques and advanced manufacturing approaches which include the current state-of-the-arts, recent developments and major accomplishments, future challenges, and directions towards fabrication of 3D *in-vitro* human tissue models, specifically in large-scale cell manufacturing and advanced manufacturing platforms.

There is a total of 6 published articles in this Research Topic: 2 review papers and 4 original research papers. One of the review papers is on large-scale cell manufacturing and it introduced a new multiple-use aseptic connector that can act as potential replacement for two main types of commonly-used devices for small volume fluid transfers (single-use sterile connectors and tube welders) in cell therapy manufacturing. The review paper highlighted that multiple-use aseptic connector can fulfil the unmet need for a sterile connector suitable for small volume fluid transfers and reduce the footprint, complexity and cost of culture systems (Wu et al.). The next review paper is on advanced manufacturing platforms and it discussed how the emergence and development of smart metamaterial, advanced optimization algorithm and advanced manufacturing technique have resulted in a paradigm shift in the design, fabrication and characterization of bone scaffolds (Huo et al.). The review paper provided detailed information on the design of microstructure of the bone scaffold, application of metamaterial in the design of bone scaffolds and optimization of the microstructure in bone scaffolds, the advanced manufacturing of bone scaffolds and lastly the various techniques used for evaluating the performance of bone scaffolds.

Next, the 4 original research papers are related to the advanced manufacturing platforms. One of the original research papers reported a versatile cell-friendly photopolymerization approach that facilitated single-step fabrication of hollow-core and solid-core hydrogel fibres loaded with living cells (Savelyev et al.). The approach was implemented by extruding cell-laden hyaluronic acid glycidyl methacrylate hydrogel directly into an aqueous solution containing free radicals generated by continuous blue light photo-excitation of the flavin mononucleotide/triethanolamine photo-initiator to induce diffusion-limited photo-fabrication. The next original research paper reported the fabrication of affordable, flexible and highly-reproducible 3D bioprinted colorectal cancer model (Sbirkov et al.). The fabricated 3D colorectal cancer models exhibited greater pathomorphological resemblance to tumours and increased overall resistance to commonly used chemotherapeutics as compared to 2D cell cultures. Hence, the study has reported a novel accessible 3D tissue model platform for disease modelling and drug testing. Another original research paper demonstrated the potential of micro-vascularized skin-on-a-chip tissue equivalents for systematic delivery of therapeutics (Jones et al.). The novel vascularized skin-on-a-chip model consisted of human-derived primary and immortalized cells (pericyte co-cultures); the results indicated that vascularization enhanced the stratification and differentiation of the epidermis to form matured skin equivalents in microfluidic chips. The last original research paper demonstrated the fabrication of personalized 3D-printed bioresorbable airway external splint for sever tracheomalacia (Yu et al.). The study evaluated the performance of 3D-printed bioresorbable airway external splint on nine different young patients with severe tracheomalacia and the results showed that the 3D printed splint not only limited the external compression and prevented airway collapse but also ensured the growth potential of the airway, making it a safe, reliable, and effective treatment for congenital heart disease patients with tracheomalacia.

Numerous studies have shown that the use of conventional 2D cell culture is unable to adequately recapitulate important *in-vivo* cell-cell and cell-matrix interactions and numerous cell types have expressed different phenotypes and genomic profiles in 2D versus 3D cell culture (Breslin and O’Driscoll, 2013; Ng et al., 2016; Laschke and Menger, 2017). The combination of advanced cell processing approaches and 3D bioprinting technology is critical for highly-reproducible automated fabrication of 3D human tissue models for various testing applications. We anticipate that insights and perspectives from this Research Topic would encourage the use of biomimetic 3D human tissue models for various drug/chemical testing applications to improve the prediction of human responses in an accurate and reliable manner.
